# A Case of Triplets

**Published:** 1841-04

**Authors:** A. H. Buchanan

**Affiliations:** Columbia, Tennessee


Art. II.—*A Case of Triplets.* By A. H. Buchanan, M. D., of Columbia, Tennessee.

In the month of September last, I was consulted by Wm. E. Ervin, Esq., concerning Mrs. E., who, he said, was very large and suffered much from pain and swelling of the lower extremities; he also expressed the fear that she was pregnant with twins from her unusual size, &c. Laxatives, light diet and rest were recommended. From this time until the 20th of October, I was occasionally consulted about her situation in consequence of more or less pain in the back, abdomen and lower extremities.

On the 24th of October a slight hæmorrhage took place from the uterus, and I was requested to visit her; on my arrival the slight hæmorrhage and pain which accompanied it had ceased, and I returned home, (distance three miles,) after advising him of the fact, however, that the position of the placenta over the mouth of the womb might probably be the cause of the sanguineous discharge, and should it return to inform me immediately. On the next morning, the uterine pain and hæmorrhage having returned, I again visited her; but as the discharge had ceased before my arrival, and the pains were very irregular and inefficient, I again left her and returned in the evening to remain with her during the night.

She passed a very restless night, suffering more or less from pain and mental anxiety, and often observing that something unusual was the matter with her. On the next morning, the pains becoming more urgent, I examined her per vaginam and found the mouth of the womb widely dilated, and the head of a child low in the pelvis; the membranes though not tense were ruptured, and, in a very few minutes, she gave birth to a fine, large boy, weighing something upwards of seven pounds. The size of this child induced me to believe that there were no more to follow, and I waited about ten minutes for the placenta, but finding the mother very languid and becoming pale, I endeavored to ascertain the condition of the womb; and upon examination found her flooding freely, and the mouth of the womb above the reach of the finger. The hand was now applied upon the abdomen, which felt as large and hard as if she had not given birth to a child. By this time she had become extremely pale and almost faint and pulseless. Brandy toddy and laudanum were ordered to be given freely and frequently. The hand was now introduced into the womb, the mouth of which was slightly contracted about a portion of the placenta which was hanging through its lips; the hand in passing into the womb first came in contact with a head, a hand and a foot, which somewhat puzzled me, for I had no thought of there being two more children. I endeavored to rupture the membranes which was somewhat difficult to accomplish, both on account of their strength and the small amount of liquor amnii they contained, rendering them loose and flabby about the child; having succeeded, however, the foot was seized and brought down to the vulva, where it was retained by a fillet of tape; the left hand was now introduced and the other foot brought down, and I was also fully satisfied of the existence of a third child. The child whose feet were down was delivered without much assistance from the womb, although I endeavored to excite contraction by external friction, &c., &c. The feet of the third child were now brought to the vulva, and brisk friction over the abdomen to excite contraction of the womb, which being accomplished to some extent, the child was delivered; it was nearly lifeless, but there being free pulsation in the cord, it was not taken from the mother until free respiration was established. I was careful at the delivery of each child to tie both ends of the cord. The condition of the mother was now extremely critical, and to ensure contraction of the womb, I kept up brisk frictions over the abdomen for half an hour or more, before I attempted the delivery of the placenta, which being accomplished, I kept up friction at intervals for half an hour longer. The mother all this time was extremely exhausted and almost pulseless; but by persevering in the use of the brandy and laudanum, and keeping up the frictions, at the end of two hours the pulse rallied and the womb contracted, and in three hours I had the satisfaction of seeing my patient safe. She threw up the brandy, when a large pill of opium was given with a very happy effect. She drank about a pint of brandy toddy and two teaspoonsful of laudanum in about two hours; she recovered without a bad symptom. The children are all well formed and still living and hearty—two males and one female. The first, a male, weighing seven pounds; second, male, four pounds; third, female, five pounds—making in all sixteen pounds. The placenta was very large, by actual measurement it was twelve inches across in one direction and fourteen in another, and two inches thick through the centre: it was one placenta with three divisions upon its fœtal surface; each child floated in its own waters and was surrounded by its own membranes, there being three distinct cords. Mrs. E. is a delicate woman, aged about thirty-five years, and the mother of seven children, including the last three. The father is a stout, healthy man of middle age.

*December* 12*th*, 1840.

